# Achieving the Triple Aim Through Informed Consent for Computed Tomography

**DOI:** 10.5811/westjem.2015.12.29466

**Published:** 2015-12-16

**Authors:** Dylan Carney, Robert M. Rodriguez

**Affiliations:** University of California, San Francisco, Department of Emergency Medicine, San Francisco, California

At the end of a particularly busy shift, you meet Mary, a 24 year-old female with no past medical history, who presents with six hours of crampy, intermittent, periumbilical abdominal pain but no associated fever, nausea, vomiting, diarrhea or anorexia. Her vital signs are normal and her abdominal and gynecological exams are notable only for mild, diffuse abdominal tenderness without rebound or guarding. Her lab results and urinalysis are unremarkable, and her pain improves somewhat with intravenous pain medications. You explain to the patient that you have a low suspicion for an intraabdominal emergency, but cannot be certain without a computed tomography (CT) scan. “I’ll do whatever you recommend,” she replies. The patient ultimately gets a CT, which is normal, and she is discharged 30 minutes later with a diagnosis of nonspecific abdominal pain.

Emergency department (ED) clinicians see patients like Mary every day- patients for whom our experience and clinical suspicion of a truly emergent condition is low, albeit not negligible, and for whom there are no consensus, evidence-based guidelines or algorithms to guide the use of advanced imaging. The decision to image is, at times, dictated by systems-factors, such as difficulties arranging for adequate follow-up. Fear of litigation has been cited as a common driver of excessive diagnostic testing.^1^ Ultimately, diagnostic CT use in the ED is on a steep rise, which, combined with unchanging prevalence of disease, results in greater exposure to CT risks and costs with lower corresponding diagnostic yield.^2^–^5^

The risks (and costs) of CT are undeniably real and should not be ignored or minimized. Extrapolating from data from atomic bomb survivors, Smith-Bindman, Brenner and others have calculated the risks of cancer development associated with CT and estimated that up to 2% of all cancers in the US are attributable to CT scans.^6^,^7^ Beyond these theoretical extrapolations, Mathews and others demonstrated a dose-response risk of cancer development associated with CT scans in a large cohort of patients in Australia.^8^

Concurring with these concerns about CT, a number of professional medical societies and various governing bodies (BEIR- The National Research Council’s Biological Effects of Ionizing Radiation VII Report, UNSCEAR- The United Nations Scientific Committee on the Effects of Atomic Radiation, and IRCP- The International Commission on Radiological Protection) have targeted cutbacks in unnecessary CT imaging and reduction of radiation doses with CT as specialty-wide goals.^9^–^12^ The American College of Surgeons and the American College of Emergency Physicians have both included reductions in CT as part of their Choosing Wisely campaigns.^13^,^14^

Merck and colleagues in this issue of Western Journal of Emergency Medicine, note the risks of CT, comparing them to the risks associated with blood transfusion (for which clinicians universally obtain informed consent).^15^ This is a thought-provoking and rational link that, combined with the fact that patients (and physicians) are unaware of CT risks, leads to the logical next-step of their novel trial of informed consent for abdominal/pelvic CT. In this multiphase, observational cohort study, the authors collected data on abdominal pain patients and built a multivariate logistic regression model to assess probability of CT utilization as a function of history, exam findings, diagnostic testing and disposition. Patients who had CT scans were included in a second multivariable model that estimated the likelihood of having a positive scan. In the next phase of the study, emergency providers used a one-page, standardized, written informed consent tool, which included potential biological risks and diagnostic benefits of CT, to engage patients in shared decision making. The authors report that the tool took less than one minute to use and was minimally disruptive to provider workflow. Patients in this implementation phase were stratified as “low” or “high” risk based on clinical factors (focal or rebound tenderness and the presence of a rigid abdomen). The investigators then built a logistic regression model to assess CT utilization among the low and high risk groups after controlling for confounders with propensity score matching.^15^

Their results were striking. While CT utilization was unaffected in the high-risk group, the investigators noted a 24% reduction in CT utilization among low-risk patients after the implementation of their written informed consent protocol. Notably, they found no difference in adverse events or patient return visits within 30 days among the nearly 4,000 patients included in the study, indicating that their protocol was both safe and effective.^15^

Overall, these findings introduce a novel way to improve patient-centered care. Their informed-consent intervention was simple, safe, fast, and effective, achieving several goals of the Institute for Health Improvement’s Triple Aims: (1) improving population health, (2) reducing costs and (3) enhancing the patient experience.^16^ Decreasing the rate of low-yield CT scans may decrease costs and improve outcomes by minimizing unnecessary ionizing radiation and decreasing potentially invasive workups of incidental findings. At least as important are the potential positive effects of shared decision-making. Prior investigators have shown that patients want to be informed of the risks (and costs) of CT whenever possible and that many patients would prefer to avoid imaging when their risk of life-threatening injury is low.^17^ Informed consent for CT may therefore improve patient satisfaction and patient care experience.

Should informed consent for CT be routinely obtained? Should it even become a standard of care? Although there are certainly some cases in which consent may not be feasible, most scenarios for CT use in the ED are truly not emergent enough to preclude informed consent/shared decision-making. We await further examinations of this important topic, most notably a large, multicenter study of shared-decision making in pediatric head trauma.^18^ In the meantime, the work of Merck et al is compelling enough that we would advocate providing consent for CT in low risk abdominal pain cases.
